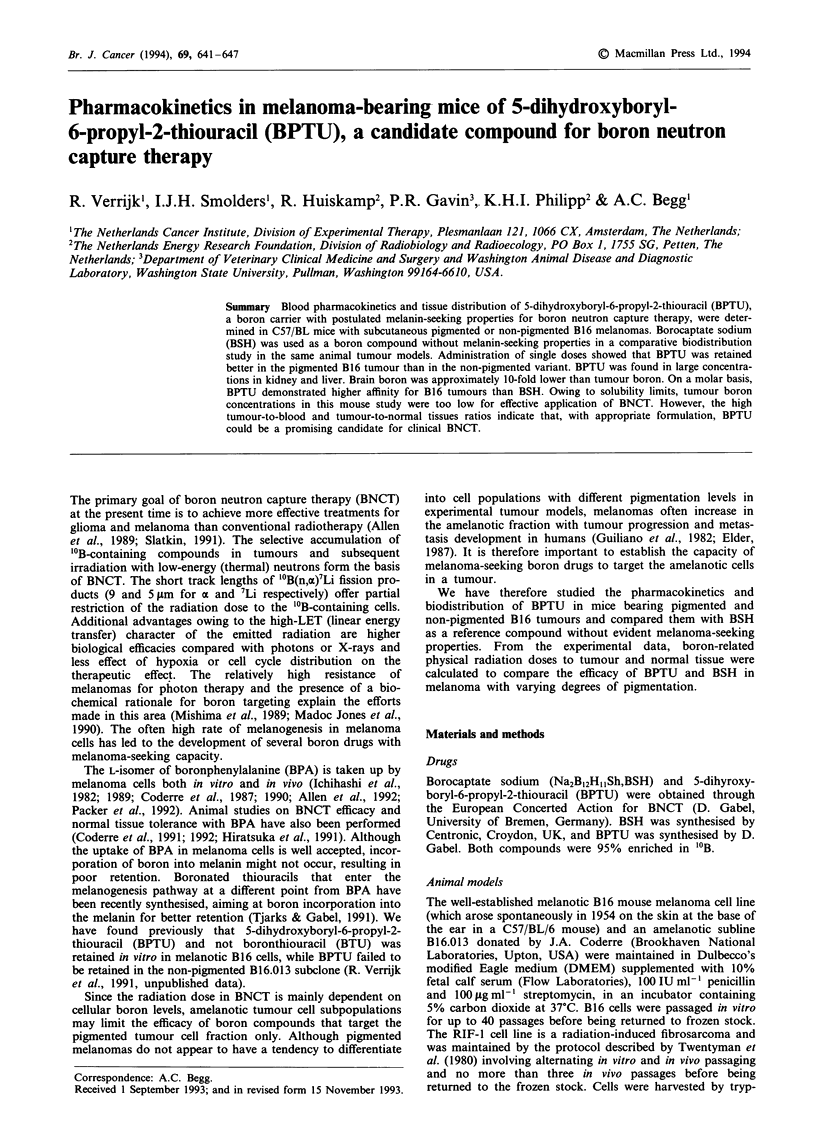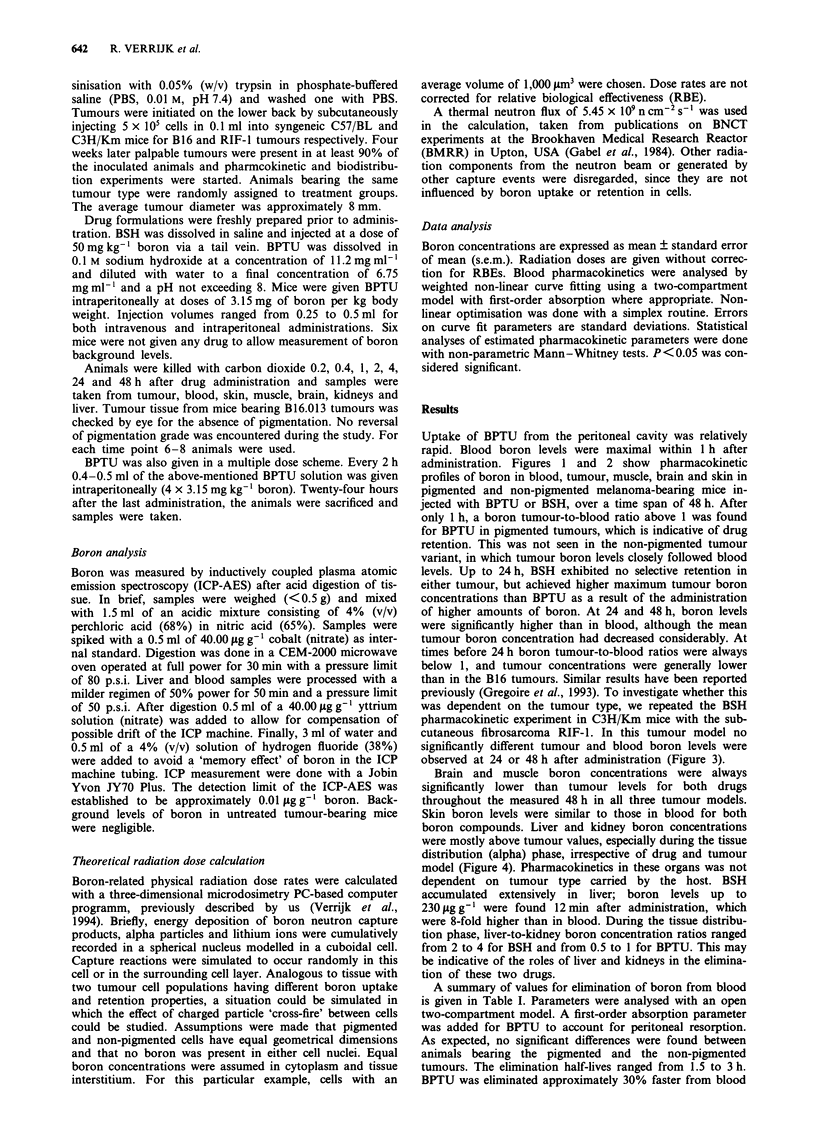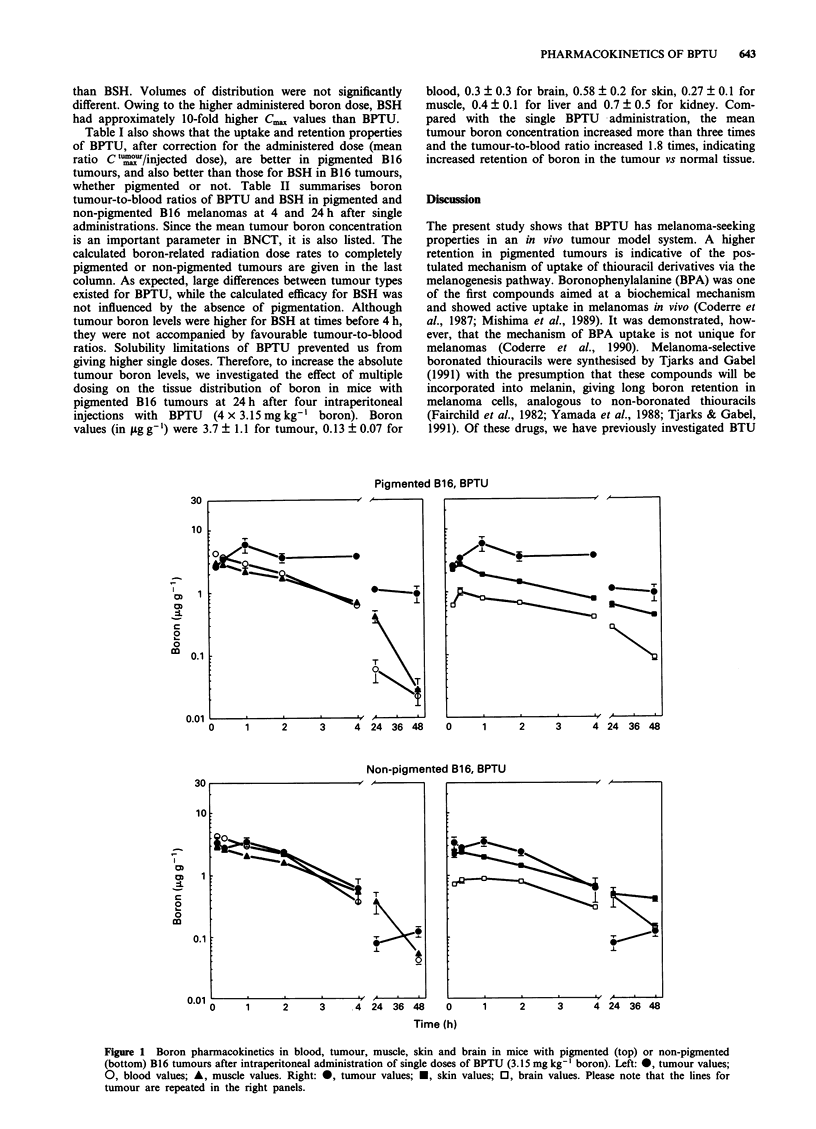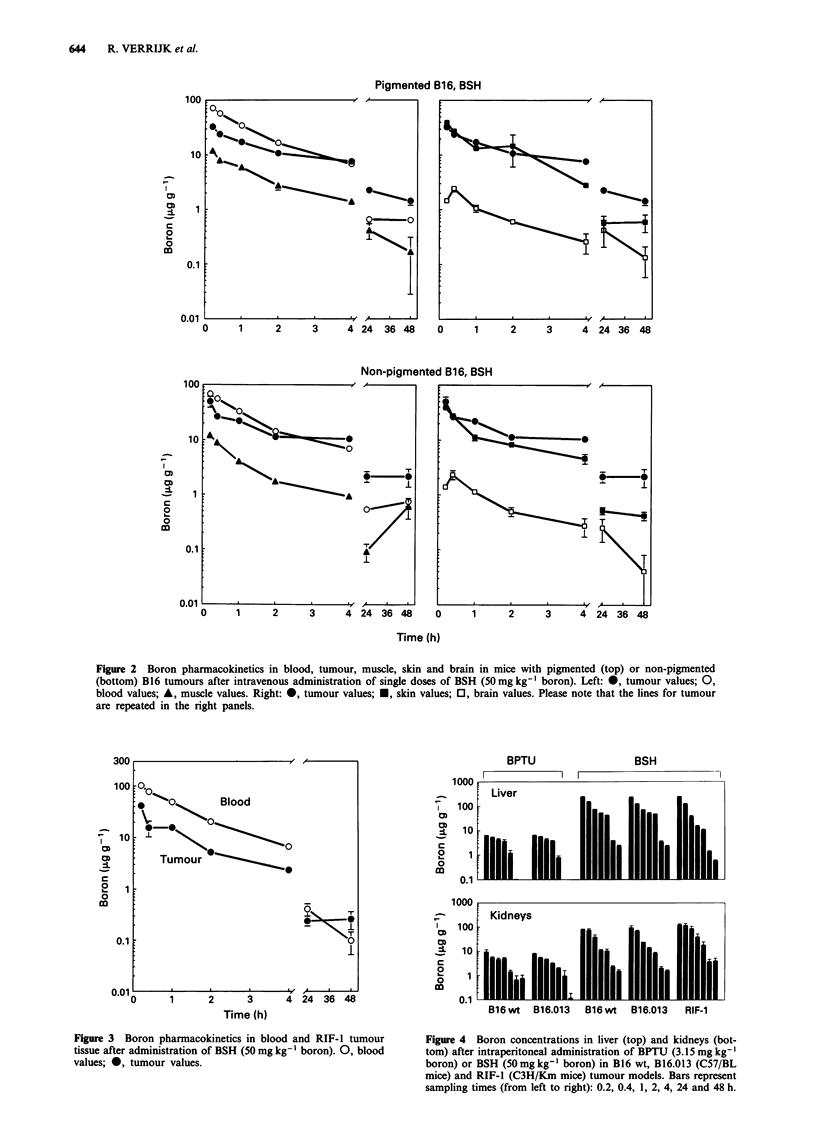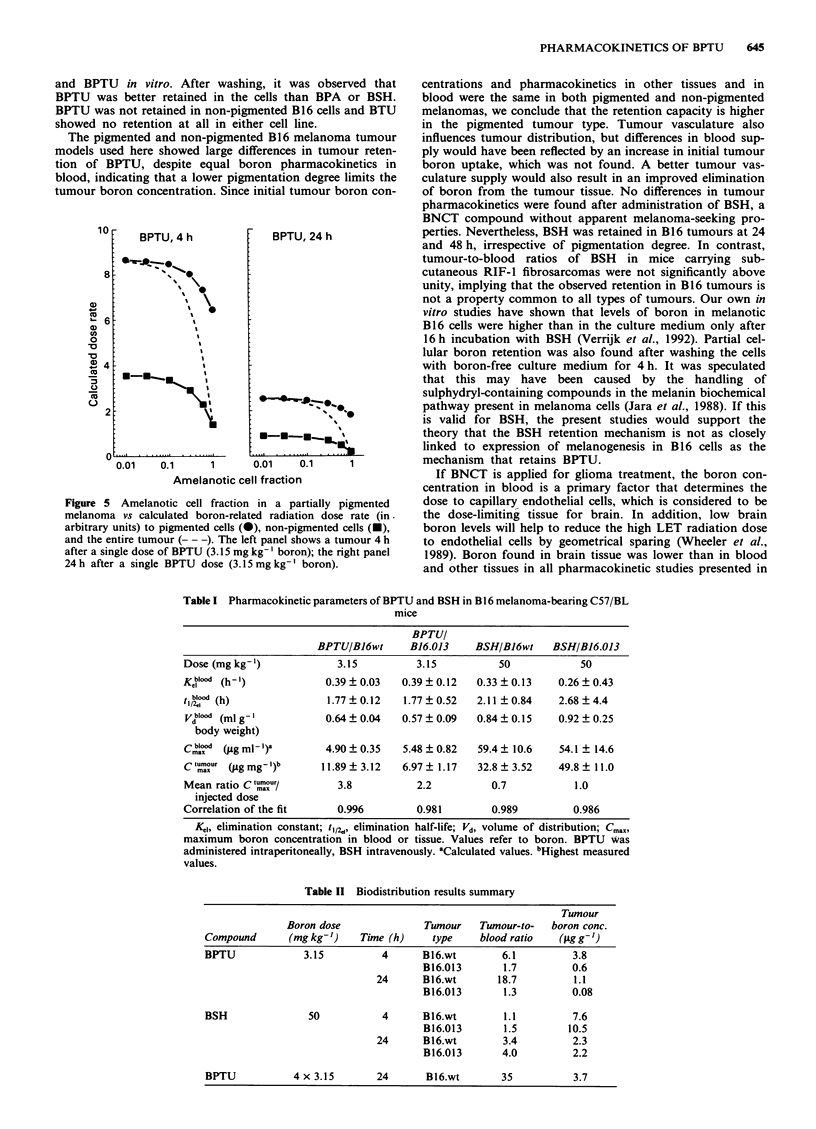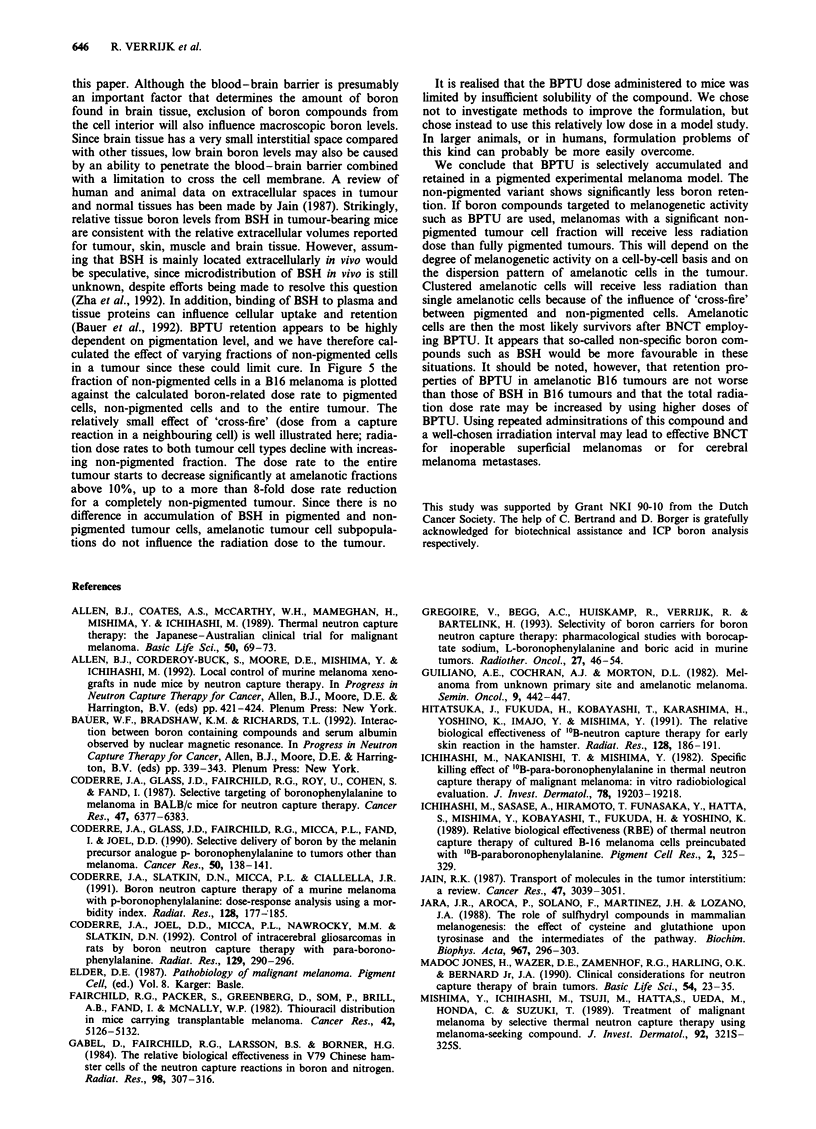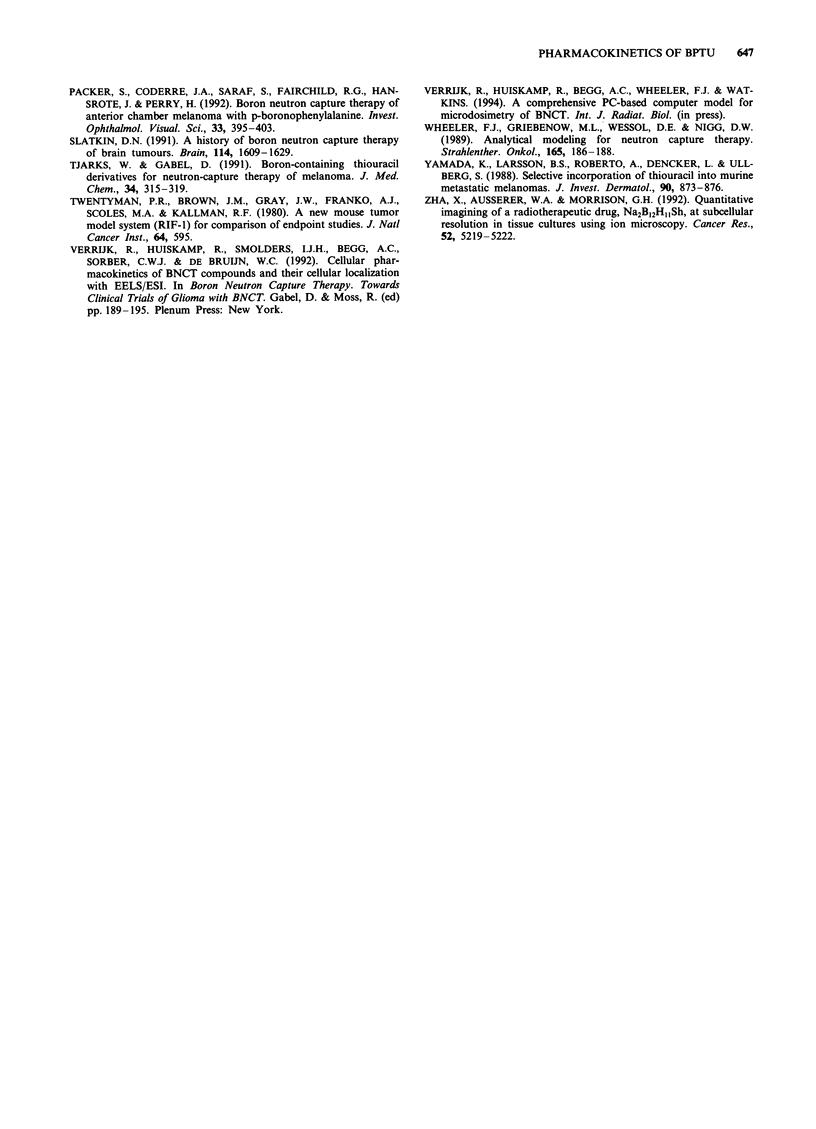# Pharmacokinetics in melanoma-bearing mice of 5-dihydroxyboryl-6-propyl-2-thiouracil (BPTU), a candidate compound for boron neutron capture therapy.

**DOI:** 10.1038/bjc.1994.125

**Published:** 1994-04

**Authors:** R. Verrijk, I. J. Smolders, R. Huiskamp, P. R. Gavin, K. H. Philipp, A. C. Begg

**Affiliations:** The Netherlands Cancer Institute, Division of Experimental Therapy, Amsterdam.

## Abstract

Blood pharmacokinetics and tissue distribution of 5-dihydroxyboryl-6-propyl-2-thiouracil (BPTU), a boron carrier with postulated melanin-seeking properties for boron neutron capture therapy, were determined in C57/BL mice with subcutaneous pigmented or non-pigmented B16 melanomas. Borocaptate sodium (BSH) was used as a boron compound without melanin-seeking properties in a comparative biodistribution study in the same animal tumour models. Administration of single doses showed that BPTU was retained better in the pigmented B16 tumour than in the non-pigmented variant. BPTU was found in large concentrations in kidney and liver. Brain boron was approximately 10-fold lower than tumour boron. On a molar basis, BPTU demonstrated higher affinity for B16 tumours than BSH. Owing to solubility limits, tumour boron concentrations in this mouse study were too low for effective application of BNCT. However, the high tumour-to-blood and tumour-to-normal tissues ratios indicate that, with appropriate formulation, BPTU could be a promising candidate for clinical BNCT.


Br. J. Cancer (1994), 69, 641–647 © Macmillan Press Ltd., 1994

### Pharmacokinetics in melanoma-bearing mice of 5-dihydroxyboryl-6-propyl-2-thiouracil (BPTU), a candidate compound for boron neutron capture therapy

R. Verrijk[1], I.J.H. Smolders[1], R. Huiskamp[2], P.R. Gavin[3], K.H.I. Philipp[2] & A.C. Begg[1]

[1]*The Netherlands Cancer Institute, Division of Experimental Therapy, Plesmanlaan 121, 1066 CX, Amsterdam, The Netherlands;* [2]*The Netherlands Energy Research Foundation, Division of Radiobiology and Radioecology, PO Box 1, 1755 SG, Petten, The Netherlands;* [3]*Department of Veterinary Clinical Medicine and Surgery and Washington Animal Disease and Diagnostic Laboratory, Washington State University, Pullman, Washington 99164-6610, USA.*

**Summary** Blood pharmacokinetics and tissue distribution of 5-dihydroxyboryl-6-propyl-2-thiouracil (BPTU), a boron carrier with postulated melanin-seeking properties for boron neutron capture therapy, were determined in C57/BL mice with subcutaneous pigmented or non-pigmented B16 melanomas. Borocaptate sodium (BSH) was used as a boron compound without melanin-seeking properties in a comparative biodistribution study in the same animal tumour models. Administration of single doses showed that BPTU was retained better in the pigmented B16 tumour than in the non-pigmented variant. BPTU was found in large concentrations in kidney and liver. Brain boron was approximately 10-fold lower than tumour boron. On a molar basis, BPTU demonstrated higher affinity for B16 tumours than BSH. Owing to solubility limits, tumour boron concentrations in this mouse study were too low for effective application of BNCT. However, the high tumour-to-blood and tumour-to-normal tissues ratios indicate that, with appropriate formulation, BPTU could be a promising candidate for clinical BNCT.

The primary goal of boron neutron capture therapy (BNCT) at the present time is to achieve more effective treatments for glioma and melanoma than conventional radiotherapy (Allen *et al.*, 1989; Slatkin, 1991). The selective accumulation of $^{10}B$-containing compounds in tumours and subsequent irradiation with low-energy (thermal) neutrons form the basis of BNCT. The short track lengths of $^{10}B(n,\alpha)^{7}Li$ fission products (9 and 5 μm for α and $^{7}Li$ respectively) offer partial restriction of the radiation dose to the $^{10}B$-containing cells. Additional advantages owing to the high-LET (linear energy transfer) character of the emitted radiation are higher biological efficacies compared with photons or X-rays and less effect of hypoxia or cell cycle distribution on the therapeutic effect. The relatively high resistance of melanomas for photon therapy and the presence of a biochemical rationale for boron targeting explain the efforts made in this area (Mishima *et al.*, 1989; Madoc Jones *et al.*, 1990). The often high rate of melanogenesis in melanoma cells has led to the development of several boron drugs with melanoma-seeking capacity.

The L-isomer of boronphenylalanine (BPA) is taken up by melanoma cells both *in vitro* and *in vivo* (Ichihashi *et al.*, 1982; 1989; Coderre *et al.*, 1987; 1990; Allen *et al.*, 1992; Packer *et al.*, 1992). Animal studies on BNCT efficacy and normal tissue tolerance with BPA have also been performed (Coderre *et al.*, 1991; 1992; Hiratsuka *et al.*, 1991). Although the uptake of BPA in melanoma cells is well accepted, incorporation of boron into melanin might not occur, resulting in poor retention. Boronated thiouracils that enter the melanogenesis pathway at a different point from BPA have been recently synthesised, aiming at boron incorporation into the melanin for better retention (Tjarks & Gabel, 1991). We have found previously that 5-dihydroxyboryl-6-propyl-2-thiouracil (BPTU) and not boronthiouracil (BTU) was retained *in vitro* in melanotic B16 cells, while BPTU failed to be retained in the non-pigmented B16.013 subclone (R. Verrijk *et al.*, 1991, unpublished data).

Since the radiation dose in BNCT is mainly dependent on cellular boron levels, amelanotic tumour cell subpopulations may limit the efficacy of boron compounds that target the pigmented tumour cell fraction only. Although pigmented melanomas do not appear to have a tendency to differentiate into cell populations with different pigmentation levels in experimental tumour models, melanomas often increase in the amelanotic fraction with tumour progression and metastasis development in humans (Guiliano *et al.*, 1982; Elder, 1987). It is therefore important to establish the capacity of melanoma-seeking boron drugs to target the amelanotic cells in a tumour.

We have therefore studied the pharmacokinetics and biodistribution of BPTU in mice bearing pigmented and non-pigmented B16 tumours and compared them with BSH as a reference compound without evident melanoma-seeking properties. From the experimental data, boron-related physical radiation doses to tumour and normal tissue were calculated to compare the efficacy of BPTU and BSH in melanoma with varying degrees of pigmentation.

### Materials and methods

### *Drugs*

Borocaptate sodium ($Na_2B_{12}H_{11}Sh$,BSH) and 5-dihyroxy-boryl-6-propyl-2-thiouracil (BPTU) were obtained through the European Concerted Action for BNCT (D. Gabel, University of Bremen, Germany). BSH was synthesised by Centronic, Croydon, UK, and BPTU was synthesised by D. Gabel. Both compounds were 95% enriched in $^{10}B$.

### *Animal models*

The well-established melanotic B16 mouse melanoma cell line (which arose spontaneously in 1954 on the skin at the base of the ear in a C57/BL/6 mouse) and an amelanotic subline B16.013 donated by J.A. Coderre (Brookhaven National Laboratories, Upton, USA) were maintained in Dulbecco's modified Eagle medium (DMEM) supplemented with 10% fetal calf serum (Flow Laboratories), 100 IU $ml^{-1}$ penicillin and 100 μg $ml^{-1}$ streptomycin, in an incubator containing 5% carbon dioxide at 37°C. B16 cells were passaged *in vitro* for up to 40 passages before being returned to frozen stock. The RIF-1 cell line is a radiation-induced fibrosarcoma and was maintained by the protocol described by Twentyman *et al.* (1980) involving alternating *in vitro* and *in vivo* passaging and no more than three *in vivo* passages before being returned to the frozen stock. Cells were harvested by tryp-

Correspondence: A.C. Begg.
Received 1 September 1993; and in revised form 15 November 1993.

sinisation with 0.05% (w/v) trypsin in phosphate-buffered saline (PBS, 0.01 M, pH 7.4) and washed one with PBS. Tumours were initiated on the lower back by subcutaneously injecting $5 \times 10^5$ cells in 0.1 ml into syngeneic C57/BL and C3H/Km mice for B16 and RIF-1 tumours respectively. Four weeks later palpable tumours were present in at least 90% of the inoculated animals and pharmcokinetic and biodistribution experiments were started. Animals bearing the same tumour type were randomly assigned to treatment groups. The average tumour diameter was approximately 8 mm.

Drug formulations were freshly prepared prior to administration. BSH was dissolved in saline and injected at a dose of 50 mg $kg^{-1}$ boron via a tail vein. BPTU was dissolved in 0.1 M sodium hydroxide at a concentration of 11.2 mg $ml^{-1}$ and diluted with water to a final concentration of 6.75 mg $ml^{-1}$ and a pH not exceeding 8. Mice were given BPTU intraperitoneally at doses of 3.15 mg of boron per kg body weight. Injection volumes ranged from 0.25 to 0.5 ml for both intravenous and intraperitoneal administrations. Six mice were not given any drug to allow measurement of boron background levels.

Animals were killed with carbon dioxide 0.2, 0.4, 1, 2, 4, 24 and 48 h after drug administration and samples were taken from tumour, blood, skin, muscle, brain, kidneys and liver. Tumour tissue from mice bearing B16.013 tumours was checked by eye for the absence of pigmentation. No reversal of pigmentation grade was encountered during the study. For each time point 6–8 animals were used.

BPTU was also given in a multiple dose scheme. Every 2 h 0.4–0.5 ml of the above-mentioned BPTU solution was given intraperitoneally ($4 \times 3.15$ mg $kg^{-1}$ boron). Twenty-four hours after the last administration, the animals were sacrificed and samples were taken.

*Boron analysis*

Boron was measured by inductively coupled plasma atomic emission spectroscopy (ICP-AES) after acid digestion of tissue. In brief, samples were weighed (<0.5 g) and mixed with 1.5 ml of an acidic mixture consisting of 4% (v/v) perchloric acid (68%) in nitric acid (65%). Samples were spiked with a 0.5 ml of 40.00 $\mu g\ g^{-1}$ cobalt (nitrate) as internal standard. Digestion was done in a CEM-2000 microwave oven operated at full power for 30 min with a pressure limit of 80 p.s.i. Liver and blood samples were processed with a milder regimen of 50% power for 50 min and a pressure limit of 50 p.s.i. After digestion 0.5 ml of a 40.00 $\mu g\ g^{-1}$ yttrium solution (nitrate) was added to allow for compensation of possible drift of the ICP machine. Finally, 3 ml of water and 0.5 ml of a 4% (v/v) solution of hydrogen fluoride (38%) were added to avoid a 'memory effect' of boron in the ICP machine tubing. ICP measurement were done with a Jobin Yvon JY70 Plus. The detection limit of the ICP-AES was established to be approximately 0.01 $\mu g\ g^{-1}$ boron. Background levels of boron in untreated tumour-bearing mice were negligible.

*Theoretical radiation dose calculation*

Boron-related physical radiation dose rates were calculated with a three-dimensional microdosimetry PC-based computer programm, previously described by us (Verrijk et al., 1994). Briefly, energy deposition of boron neutron capture products, alpha particles and lithium ions were cumulatively recorded in a spherical nucleus modelled in a cuboidal cell. Capture reactions were simulated to occur randomly in this cell or in the surrounding cell layer. Analogous to tissue with two tumour cell populations having different boron uptake and retention properties, a situation could be simulated in which the effect of charged particle 'cross-fire' between cells could be studied. Assumptions were made that pigmented and non-pigmented cells have equal geometrical dimensions and that no boron was present in either cell nuclei. Equal boron concentrations were assumed in cytoplasm and tissue interstitium. For this particular example, cells with an average volume of 1,000 $\mu m^3$ were chosen. Dose rates are not corrected for relative biological effectiveness (RBE).

A thermal neutron flux of $5.45 \times 10^9$ n $cm^{-2}$ $s^{-1}$ was used in the calculation, taken from publications on BNCT experiments at the Brookhaven Medical Research Reactor (BMRR) in Upton, USA (Gabel et al., 1984). Other radiation components from the neutron beam or generated by other capture events were disregarded, since they are not influenced by boron uptake or retention in cells.

*Data analysis*

Boron concentrations are expressed as mean ± standard error of mean (s.e.m.). Radiation doses are given without correction for RBEs. Blood pharmacokinetics were analysed by weighted non-linear curve fitting using a two-compartment model with first-order absorption where appropriate. Non-linear optimisation was done with a simplex routine. Errors on curve fit parameters are standard deviations. Statistical analyses of estimated pharmacokinetic parameters were done with non-parametric Mann–Whitney tests. $P < 0.05$ was considered significant.

### Results

Uptake of BPTU from the peritoneal cavity was relatively rapid. Blood boron levels were maximal within 1 h after administration. Figures 1 and 2 show pharmacokinetic profiles of boron in blood, tumour, muscle, brain and skin in pigmented and non-pigmented melanoma-bearing mice injected with BPTU or BSH, over a time span of 48 h. After only 1 h, a boron tumour-to-blood ratio above 1 was found for BPTU in pigmented tumours, which is indicative of drug retention. This was not seen in the non-pigmented tumour variant, in which tumour boron levels closely followed blood levels. Up to 24 h, BSH exhibited no selective retention in either tumour, but achieved higher maximum tumour boron concentrations than BPTU as a result of the administration of higher amounts of boron. At 24 and 48 h, boron levels were significantly higher than in blood, although the mean tumour boron concentration had decreased considerably. At times before 24 h boron tumour-to-blood ratios were always below 1, and tumour concentrations were generally lower than in the B16 tumours. Similar results have been reported previously (Gregoire et al., 1993). To investigate whether this was dependent on the tumour type, we repeated the BSH pharmacokinetic experiment in C3H/Km mice with the subcutaneous fibrosarcoma RIF-1. In this tumour model no significantly different tumour and blood boron levels were observed at 24 or 48 h after administration (Figure 3).

Brain and muscle boron concentrations were always significantly lower than tumour levels for both drugs throughout the measured 48 h in all three tumour models. Skin boron levels were similar to those in blood for both boron compounds. Liver and kidney boron concentrations were mostly above tumour values, especially during the tissue distribution (alpha) phase, irrespective of drug and tumour model (Figure 4). Pharmacokinetics in these organs was not dependent on tumour type carried by the host. BSH accumulated extensively in liver; boron levels up to 230 $\mu g\ g^{-1}$ were found 12 min after administration, which were 8-fold higher than in blood. During the tissue distribution phase, liver-to-kidney boron concentration ratios ranged from 2 to 4 for BSH and from 0.5 to 1 for BPTU. This may be indicative of the roles of liver and kidneys in the elimination of these two drugs.

A summary of values for elimination of boron from blood is given in Table I. Parameters were analysed with an open two-compartment model. A first-order absorption parameter was added for BPTU to account for peritoneal resorption. As expected, no significant differences were found between animals bearing the pigmented and the non-pigmented tumours. The elimination half-lives ranged from 1.5 to 3 h. BPTU was eliminated approximately 30% faster from blood than BSH. Volumes of distribution were not significantly different. Owing to the higher administered boron dose, BSH had approximately 10-fold higher $C_{max}$ values than BPTU.

Table I also shows that the uptake and retention properties of BPTU, after correction for the administered dose (mean ratio $C^{tumour}_{max}$/injected dose), are better in pigmented B16 tumours, and also better than those for BSH in B16 tumours, whether pigmented or not. Table II summarises boron tumour-to-blood ratios of BPTU and BSH in pigmented and non-pigmented B16 melanomas at 4 and 24 h after single administrations. Since the mean tumour boron concentration is an important parameter in BNCT, it is also listed. The calculated boron-related radiation dose rates to completely pigmented or non-pigmented tumours are given in the last column. As expected, large differences between tumour types existed for BPTU, while the calculated efficacy for BSH was not influenced by the absence of pigmentation. Although tumour boron levels were higher for BSH at times before 4 h, they were not accompanied by favourable tumour-to-blood ratios. Solubility limitations of BPTU prevented us from giving higher single doses. Therefore, to increase the absolute tumour boron levels, we investigated the effect of multiple dosing on the tissue distribution of boron in mice with pigmented B16 tumours at 24 h after four intraperitoneal injections with BPTU ($4 \times 3.15$ mg kg$^{-1}$ boron). Boron values (in $\mu$g g$^{-1}$) were $3.7 \pm 1.1$ for tumour, $0.13 \pm 0.07$ for blood, $0.3 \pm 0.3$ for brain, $0.58 \pm 0.2$ for skin, $0.27 \pm 0.1$ for muscle, $0.4 \pm 0.1$ for liver and $0.7 \pm 0.5$ for kidney. Compared with the single BPTU administration, the mean tumour boron concentration increased more than three times and the tumour-to-blood ratio increased 1.8 times, indicating increased retention of boron in the tumour *vs* normal tissue.

### Discussion

The present study shows that BPTU has melanoma-seeking properties in an *in vivo* tumour model system. A higher retention in pigmented tumours is indicative of the postulated mechanism of uptake of thiouracil derivatives via the melanogenesis pathway. Boronophenylalanine (BPA) was one of the first compounds aimed at a biochemical mechanism and showed active uptake in melanomas *in vivo* (Coderre *et al.*, 1987; Mishima *et al.*, 1989). It was demonstrated, however, that the mechanism of BPA uptake is not unique for melanomas (Coderre *et al.*, 1990). Melanoma-selective boronated thiouracils were synthesised by Tjarks and Gabel (1991) with the presumption that these compounds will be incorporated into melanin, giving long boron retention in melanoma cells, analogous to non-boronated thiouracils (Fairchild *et al.*, 1982; Yamada *et al.*, 1988; Tjarks & Gabel, 1991). Of these drugs, we have previously investigated BTU

**Figure 1** Boron pharmacokinetics in blood, tumour, muscle, skin and brain in mice with pigmented (top) or non-pigmented (bottom) B16 tumours after intraperitoneal administration of single doses of BPTU (3.15 mg kg$^{-1}$ boron). Left: ●, tumour values; ○, blood values; ▲, muscle values. Right: ●, tumour values; ■, skin values; □, brain values. Please note that the lines for tumour are repeated in the right panels.

**Figure 2** Boron pharmacokinetics in blood, tumour, muscle, skin and brain in mice with pigmented (top) or non-pigmented (bottom) B16 tumours after intravenous administration of single doses of BSH (50 mg $kg^{-1}$ boron). Left: ●, tumour values; ○, blood values; ▲, muscle values. Right: ●, tumour values; ■, skin values; □, brain values. Please note that the lines for tumour are repeated in the right panels.

**Figure 3** Boron pharmacokinetics in blood and RIF-1 tumour tissue after administration of BSH (50 mg $kg^{-1}$ boron). ○, blood values; ●, tumour values.

**Figure 4** Boron concentrations in liver (top) and kidneys (bottom) after intraperitoneal administration of BPTU (3.15 mg $kg^{-1}$ boron) or BSH (50 mg $kg^{-1}$ boron) in B16 wt, B16.013 (C57/BL mice) and RIF-1 (C3H/Km mice) tumour models. Bars represent sampling times (from left to right): 0.2, 0.4, 1, 2, 4, 24 and 48 h.

and BPTU *in vitro*. After washing, it was observed that BPTU was better retained in the cells than BPA or BSH. BPTU was not retained in non-pigmented B16 cells and BTU showed no retention at all in either cell line.

The pigmented and non-pigmented B16 melanoma tumour models used here showed large differences in tumour retention of BPTU, despite equal boron pharmacokinetics in blood, indicating that a lower pigmentation degree limits the tumour boron concentration. Since initial tumour boron concentrations and pharmacokinetics in other tissues and in blood were the same in both pigmented and non-pigmented melanomas, we conclude that the retention capacity is higher in the pigmented tumour type. Tumour vasculature also influences tumour distribution, but differences in blood supply would have been reflected by an increase in initial tumour boron uptake, which was not found. A better tumour vasculature supply would also result in an improved elimination of boron from the tumour tissue. No differences in tumour pharmacokinetics were found after administration of BSH, a BNCT compound without apparent melanoma-seeking properties. Nevertheless, BSH was retained in B16 tumours at 24 and 48 h, irrespective of pigmentation degree. In contrast, tumour-to-blood ratios of BSH in mice carrying subcutaneous RIF-1 fibrosarcomas were not significantly above unity, implying that the observed retention in B16 tumours is not a property common to all types of tumours. Our own *in vitro* studies have shown that levels of boron in melanotic B16 cells were higher than in the culture medium only after 16 h incubation with BSH (Verrijk *et al.*, 1992). Partial cellular boron retention was also found after washing the cells with boron-free culture medium for 4 h. It was speculated that this may have been caused by the handling of sulphydryl-containing compounds in the melanin biochemical pathway present in melanoma cells (Jara *et al.*, 1988). If this is valid for BSH, the present studies would support the theory that the BSH retention mechanism is not as closely linked to expression of melanogenesis in B16 cells as the mechanism that retains BPTU.

If BNCT is applied for glioma treatment, the boron concentration in blood is a primary factor that determines the dose to capillary endothelial cells, which is considered to be the dose-limiting tissue for brain. In addition, low brain boron levels will help to reduce the high LET radiation dose to endothelial cells by geometrical sparing (Wheeler *et al.*, 1989). Boron found in brain tissue was lower than in blood and other tissues in all pharmacokinetic studies presented in

**Figure 5** Amelanotic cell fraction in a partially pigmented melanoma *vs* calculated boron-related radiation dose rate (in arbitrary units) to pigmented cells (●), non-pigmented cells (■), and the entire tumour (- - -). The left panel shows a tumour 4 h after a single dose of BPTU (3.15 mg $kg^{-1}$ boron); the right panel 24 h after a single BPTU dose (3.15 mg $kg^{-1}$ boron).

**Table I** Pharmacokinetic parameters of BPTU and BSH in B16 melanoma-bearing C57/BL mice

| | *BPTU/B16wt* | *BPTU/ B16.013* | *BSH/B16wt* | *BSH/B16.013* |
|---|---|---|---|---|
| Dose (mg $kg^{-1}$) | 3.15 | 3.15 | 50 | 50 |
| $K_{el}^{blood}$ ($h^{-1}$) | 0.39 ± 0.03 | 0.39 ± 0.12 | 0.33 ± 0.13 | 0.26 ± 0.43 |
| $t_{1/2_{el}}^{blood}$ (h) | 1.77 ± 0.12 | 1.77 ± 0.52 | 2.11 ± 0.84 | 2.68 ± 4.4 |
| $V_d^{blood}$ (ml $g^{-1}$ body weight) | 0.64 ± 0.04 | 0.57 ± 0.09 | 0.84 ± 0.15 | 0.92 ± 0.25 |
| $C_{max}^{blood}$ (μg $ml^{-1}$)[a] | 4.90 ± 0.35 | 5.48 ± 0.82 | 59.4 ± 10.6 | 54.1 ± 14.6 |
| $C_{max}^{tumour}$ (μg $mg^{-1}$)[b] | 11.89 ± 3.12 | 6.97 ± 1.17 | 32.8 ± 3.52 | 49.8 ± 11.0 |
| Mean ratio $C_{max}^{tumour}$/ injected dose | 3.8 | 2.2 | 0.7 | 1.0 |
| Correlation of the fit | 0.996 | 0.981 | 0.989 | 0.986 |

$K_{el}$, elimination constant; $t_{1/2_{el}}$, elimination half-life; $V_d$, volume of distribution; $C_{max}$, maximum boron concentration in blood or tissue. Values refer to boron. BPTU was administered intraperitoneally, BSH intravenously. [a]Calculated values. [b]Highest measured values.

**Table II** Biodistribution results summary

| *Compound* | *Boron dose (mg $kg^{-1}$)* | *Time (h)* | *Tumour type* | *Tumour-to-blood ratio* | *Tumour boron conc. (μg $g^{-1}$)* |
|---|---|---|---|---|---|
| BPTU | 3.15 | 4 | B16.wt | 6.1 | 3.8 |
| | | | B16.013 | 1.7 | 0.6 |
| | | 24 | B16.wt | 18.7 | 1.1 |
| | | | B16.013 | 1.3 | 0.08 |
| BSH | 50 | 4 | B16.wt | 1.1 | 7.6 |
| | | | B16.013 | 1.5 | 10.5 |
| | | 24 | B16.wt | 3.4 | 2.3 |
| | | | B16.013 | 4.0 | 2.2 |
| BPTU | 4 × 3.15 | 24 | B16.wt | 35 | 3.7 |

this paper. Although the blood–brain barrier is presumably an important factor that determines the amount of boron found in brain tissue, exclusion of boron compounds from the cell interior will also influence macroscopic boron levels. Since brain tissue has a very small interstitial space compared with other tissues, low brain boron levels may also be caused by an ability to penetrate the blood–brain barrier combined with a limitation to cross the cell membrane. A review of human and animal data on extracellular spaces in tumour and normal tissues has been made by Jain (1987). Strikingly, relative tissue boron levels from BSH in tumour-bearing mice are consistent with the relative extracellular volumes reported for tumour, skin, muscle and brain tissue. However, assuming that BSH is mainly located extracellularly *in vivo* would be speculative, since microdistribution of BSH *in vivo* is still unknown, despite efforts being made to resolve this question (Zha *et al.*, 1992). In addition, binding of BSH to plasma and tissue proteins can influence cellular uptake and retention (Bauer *et al.*, 1992). BPTU retention appears to be highly dependent on pigmentation level, and we have therefore calculated the effect of varying fractions of non-pigmented cells in a tumour since these could limit cure. In Figure 5 the fraction of non-pigmented cells in a B16 melanoma is plotted against the calculated boron-related dose rate to pigmented cells, non-pigmented cells and to the entire tumour. The relatively small effect of 'cross-fire' (dose from a capture reaction in a neighbouring cell) is well illustrated here; radiation dose rates to both tumour cell types decline with increasing non-pigmented fraction. The dose rate to the entire tumour starts to decrease significantly at amelanotic fractions above 10%, up to a more than 8-fold dose rate reduction for a completely non-pigmented tumour. Since there is no difference in accumulation of BSH in pigmented and non-pigmented tumour cells, amelanotic tumour cell subpopulations do not influence the radiation dose to the tumour.

It is realised that the BPTU dose administered to mice was limited by insufficient solubility of the compound. We chose not to investigate methods to improve the formulation, but chose instead to use this relatively low dose in a model study. In larger animals, or in humans, formulation problems of this kind can probably be more easily overcome.

We conclude that BPTU is selectively accumulated and retained in a pigmented experimental melanoma model. The non-pigmented variant shows significantly less boron retention. If boron compounds targeted to melanogenetic activity such as BPTU are used, melanomas with a significant non-pigmented tumour cell fraction will receive less radiation dose than fully pigmented tumours. This will depend on the degree of melanogenetic activity on a cell-by-cell basis and on the dispersion pattern of amelanotic cells in the tumour. Clustered amelanotic cells will receive less radiation than single amelanotic cells because of the influence of 'cross-fire' between pigmented and non-pigmented cells. Amelanotic cells are then the most likely survivors after BNCT employing BPTU. It appears that so-called non-specific boron compounds such as BSH would be more favourable in these situations. It should be noted, however, that retention properties of BPTU in amelanotic B16 tumours are not worse than those of BSH in B16 tumours and that the total radiation dose rate may be increased by using higher doses of BPTU. Using repeated adminsitrations of this compound and a well-chosen irradiation interval may lead to effective BNCT for inoperable superficial melanomas or for cerebral melanoma metastases.

This study was supported by Grant NKI 90-10 from the Dutch Cancer Society. The help of C. Bertrand and D. Borger is gratefully acknowledged for biotechnical assistance and ICP boron analysis respectively.

### References

ALLEN, B.J., COATES, A.S., MCCARTHY, W.H., MAMEGHAN, H., MISHIMA, Y. & ICHIHASHI, M. (1989). Thermal neutron capture therapy: the Japanese–Australian clinical trial for malignant melanoma. *Basic Life Sci.*, **50**, 69–73.

ALLEN, B.J., CORDEROY-BUCK, S., MOORE, D.E., MISHIMA, Y. & ICHIHASHI, M. (1992). Local control of murine melanoma xenografts in nude mice by neutron capture therapy. In *Progress in Neutron Capture Therapy for Cancer*, Allen, B.J., Moore, D.E. & Harrington, B.V. (eds) pp. 421–424. Plenum Press: New York.

BAUER, W.F., BRADSHAW, K.M. & RICHARDS, T.L. (1992). Interaction between boron containing compounds and serum albumin observed by nuclear magnetic resonance. In *Progress in Neutron Capture Therapy for Cancer*, Allen, B.J., Moore, D.E. & Harrington, B.V. (eds) pp. 339–343. Plenum Press: New York.

CODERRE, J.A., GLASS, J.D., FAIRCHILD, R.G., ROY, U., COHEN, S. & FAND, I. (1987). Selective targeting of boronophenylalanine to melanoma in BALB/c mice for neutron capture therapy. *Cancer Res.*, **47**, 6377–6383.

CODERRE, J.A., GLASS, J.D., FAIRCHILD, R.G., MICCA, P.L., FAND, I. & JOEL, D.D. (1990). Selective delivery of boron by the melanin precursor analogue p- boronophenylalanine to tumors other than melanoma. *Cancer Res.*, **50**, 138–141.

CODERRE, J.A., SLATKIN, D.N., MICCA, P.L. & CIALLELLA, J.R. (1991). Boron neutron capture therapy of a murine melanoma with p-boronophenylalanine: dose-response analysis using a morbidity index. *Radiat. Res.*, **128**, 177–185.

CODERRE, J.A., JOEL, D.D., MICCA, P.L., NAWROCKY, M.M. & SLATKIN, D.N. (1992). Control of intracerebral gliosarcomas in rats by boron neutron capture therapy with para-boronophenylalanine. *Radiat. Res.*, **129**, 290–296.

ELDER, D.E. (1987). *Pathobiology of malignant melanoma. Pigment Cell*, (ed.) Vol. 8. Karger: Basle.

FAIRCHILD, R.G., PACKER, S., GREENBERG, D., SOM, P., BRILL, A.B., FAND, I. & MCNALLY, W.P. (1982). Thiouracil distribution in mice carrying transplantable melanoma. *Cancer Res.*, **42**, 5126–5132.

GABEL, D., FAIRCHILD, R.G., LARSSON, B.S. & BORNER, H.G. (1984). The relative biological effectiveness in V79 Chinese hamster cells of the neutron capture reactions in boron and nitrogen. *Radiat. Res.*, **98**, 307–316.

GREGOIRE, V., BEGG, A.C., HUISKAMP, R., VERRIJK, R. & BARTELINK, H. (1993). Selectivity of boron carriers for boron neutron capture therapy: pharmacological studies with borocaptate sodium, L-boronophenylalanine and boric acid in murine tumors. *Radiother. Oncol.*, **27**, 46–54.

GUILIANO, A.E., COCHRAN, A.J. & MORTON, D.L. (1982). Melanoma from unknown primary site and amelanotic melanoma. *Semin. Oncol.*, **9**, 442–447.

HITATSUKA, J., FUKUDA, H., KOBAYASHI, T., KARASHIMA, H., YOSHINO, K., IMAJO, Y. & MISHIMA, Y. (1991). The relative biological effectiveness of $^{10}$B-neutron capture therapy for early skin reaction in the hamster. *Radiat. Res.*, **128**, 186–191.

ICHIHASHI, M., NAKANISHI, T. & MISHIMA, Y. (1982). Specific killing effect of $^{10}$B-para-boronophenylalanine in thermal neutron capture therapy of malignant melanoma: in vitro radiobiological evaluation. *J. Invest. Dermatol.*, **78**, 19203–19218.

ICHIHASHI, M., SASASE, A., HIRAMOTO, T. FUNASAKA, Y., HATTA, S., MISHIMA, Y., KOBAYASHI, T., FUKUDA, H. & YOSHINO, K. (1989). Relative biological effectiveness (RBE) of thermal neutron capture therapy of cultured B-16 melanoma cells preincubated with $^{10}$B-paraboronophenylalanine. *Pigment Cell Res.*, **2**, 325–329.

JAIN, R.K. (1987). Transport of molecules in the tumor interstitium: a review. *Cancer Res.*, **47**, 3039–3051.

JARA, J.R., AROCA, P., SOLANO, F., MARTINEZ, J.H. & LOZANO, J.A. (1988). The role of sulfhydryl compounds in mammalian melanogenesis: the effect of cysteine and glutathione upon tyrosinase and the intermediates of the pathway. *Biochim. Biophys. Acta*, **967**, 296–303.

MADOC JONES, H., WAZER, D.E., ZAMENHOF, R.G., HARLING, O.K. & BERNARD Jr, J.A. (1990). Clinical considerations for neutron capture therapy of brain tumors. *Basic Life Sci.*, **54**, 23–35.

MISHIMA, Y., ICHIHASHI, M., TSUJI, M., HATTA,S., UEDA, M., HONDA, C. & SUZUKI, T. (1989). Treatment of malignant melanoma by selective thermal neutron capture therapy using melanoma-seeking compound. *J. Invest. Dermatol.*, **92**, 321S–325S.

PACKER, S., CODERRE, J.A., SARAF, S., FAIRCHILD, R.G., HANSROTE, J. & PERRY, H. (1992). Boron neutron capture therapy of anterior chamber melanoma with p-boronophenylalanine. *Invest. Ophthalmol. Visual. Sci.*, **33**, 395–403.

SLATKIN, D.N. (1991). A history of boron neutron capture therapy of brain tumours. *Brain*, **114**, 1609–1629.

TJARKS, W. & GABEL, D. (1991). Boron-containing thiouracil derivatives for neutron-capture therapy of melanoma. *J. Med. Chem.*, **34**, 315–319.

TWENTYMAN, P.R., BROWN, J.M., GRAY, J.W., FRANKO, A.J., SCOLES, M.A. & KALLMAN, R.F. (1980). A new mouse tumor model system (RIF-1) for comparison of endpoint studies. *J. Natl Cancer Inst.*, **64**, 595.

VERRIJK, R., HUISKAMP, R., SMOLDERS, I.J.H., BEGG, A.C., SORBER, C.W.J. & DE BRUIJN, W.C. (1992). Cellular pharmacokinetics of BNCT compounds and their cellular localization with EELS/ESI. In *Boron Neutron Capture Therapy. Towards Clinical Trials of Glioma with BNCT.* Gabel, D. & Moss, R. (ed) pp. 189–195. Plenum Press: New York.

VERRIJK, R., HUISKAMP, R., BEGG, A.C., WHEELER, F.J. & WATKINS. (1994). A comprehensive PC-based computer model for microdosimetry of BNCT. *Int. J. Radiat. Biol.* (in press).

WHEELER, F.J., GRIEBENOW, M.L., WESSOL, D.E. & NIGG, D.W. (1989). Analytical modeling for neutron capture therapy. *Strahlenther. Onkol.*, **165**, 186–188.

YAMADA, K., LARSSON, B.S., ROBERTO, A., DENCKER, L. & ULLBERG, S. (1988). Selective incorporation of thiouracil into murine metastatic melanomas. *J. Invest. Dermatol.*, **90**, 873–876.

ZHA, X., AUSSERER, W.A. & MORRISON, G.H. (1992). Quantitative imagining of a radiotherapeutic drug, $Na_2B_{12}H_{11}Sh$, at subcellular resolution in tissue cultures using ion microscopy. *Cancer Res.*, **52**, 5219–5222.